# Glutamatergic medication in the treatment of obsessive compulsive disorder (OCD) and autism spectrum disorder (ASD) – study protocol for a randomised controlled trial

**DOI:** 10.1186/s13063-016-1266-8

**Published:** 2016-03-17

**Authors:** Alexander Häge, Tobias Banaschewski, Jan K. Buitelaar, Rick M. Dijkhuizen, Barbara Franke, David J. Lythgoe, Konstantin Mechler, Steven C. R. Williams, Ralf W. Dittmann, the TACTICS Consortium

**Affiliations:** Paediatric Psychopharmacology, Department of Child and Adolescent Psychiatry and Psychotherapy, Central Institute of Mental Health, Medical Faculty Mannheim, University of Heidelberg, PO Box 12 21 20, 68072 Mannheim, Germany; Department of Child and Adolescent Psychiatry and Psychotherapy, Central Institute of Mental Health, Medical Faculty Mannheim, University of Heidelberg, Mannheim, Germany; Department of Cognitive Neuroscience, Donders Institute for Brain, Cognition and Behaviour, Radboud University Medical Center, Nijmegen, The Netherlands; Biomedical MR Imaging and Spectroscopy Group, Center for Image Sciences, University Medical Center Utrecht, Utrecht, The Netherlands; Departments of Human Genetics and Psychiatry, Donders Institute for Brain, Cognition and Behaviour, Radboud University Medical Center, Nijmegen, The Netherlands; Department of Neuroimaging, King’s College London, Institute of Psychiatry, Psychology and Neuroscience, London, UK

**Keywords:** Glutamate, Memantine, Obsessive-compulsive disorder, Autism, Pilot trial, Psychopharmacology, Child and adolescent psychiatry

## Abstract

**Background:**

Compulsivity is a cross-disorder trait underlying phenotypically distinct psychiatric disorders that emerge in childhood or adolescence. Despite the effectiveness of serotonergic compounds in the treatment of obsessive-compulsive disorder, treatment-resistant symptoms remaining in 40 to 60 % of patients present a pressing clinical problem. There are currently no medications that effectively treat the core impairments of autism spectrum disorder. There is an urgent need for the development of conceptually novel pharmacological strategies. Agents targeting glutamate neurotransmission, such as memantine, represent promising candidates. This proof-of-concept clinical study will allow pilot-testing of memantine for both clinical effectiveness and tolerability/safety. Memantine is an *N*-methyl-D-aspartate receptor antagonist, approved for the treatment of Alzheimer’s dementia in a number of countries.

**Methods/Design:**

This 12-week study has an add-on, randomised, double-blind, placebo-controlled design of treatment with memantine, including an up-titration phase (forced flexible dose design, 5–15 mg/day), in patients aged 6–17 years and 9 months with obsessive-compulsive disorder or autism spectrum disorder.

It is planned to include patients with obsessive-compulsive disorder (*N* = 50) or autism spectrum disorder (*N* = 50) across four centres in three European countries. Patients will be randomly assigned to memantine or placebo in a 1:1 ratio. Primary objectives are the investigation of the effectiveness of memantine in paediatric patients for improving symptoms of compulsivity (primary outcome measure: total score on the Children’s Yale-Brown Obsessive-Compulsive Scale) and to explore its tolerability and safety. Secondary objectives are to explore the effects of memantine at the level of structure, function and biochemistry of the fronto-striatal circuits, and to collect blood for genetic analyses and biomarkers. Tertiary objectives are to explore the role of new candidate genes and pathways for compulsivity by linking genes to clinical phenotypes, response to treatment, neurocognitive test performance, and key structural and functional neuroimaging measures of the fronto-striatal circuits and to explore biomarkers/proteomics for compulsivity traits.

**Discussion:**

This study is part of the large, translational project TACTICS (http://www.tactics-project.eu/) that is funded by the European Union and investigates the neural, genetic and molecular factors involved in the pathogenesis of compulsivity. Its results will provide clinically relevant solid information on potential new mechanisms and medication treatment in obsessive-compulsive and autism spectrum disorders.

**Trial registration:**

EudraCT Number: 2014-003080-38, date of registration: 14 July 2014.

## Background

Compulsivity is a cross-disorder trait underlying phenotypically distinct psychiatric disorders that emerge in childhood or adolescence. Compulsivity is defined as the repetitive, irresistible urge to perform a behaviour, the experience of loss of voluntary control over this intense urge, the diminished ability to delay or inhibit thoughts or behaviours, and the tendency to perform repetitive acts in a habitual or stereotyped manner [1]. Obsessive compulsive disorders (OCD) are characterised by repetitive thoughts, impulses or images (obsessions) and repetitive behaviours or mental acts (compulsions) [2] and typically have onset in late childhood or early adolescence and mid-adulthood [3, 4]. Autism spectrum disorders (ASD) are characterised by persistent deficits in (1) social communication and social interaction across multiple contexts, and by (2) restricted, repetitive patterns of behaviour, interests or activities. ASD have their onset prior to age 3 years, and a strong persistence over time in childhood, over adolescence and into adulthood [2].

This study is part of a large, translational project funded by the European Community's Seventh Framework Programme (FP7/2007-2013) under grant agreement number 278948, TACTICS (Translational Adolescent and Childhood Therapeutic Interventions in Compulsive Syndromes). It aims to identify the neural, genetic and molecular factors involved in the pathogenesis of compulsivity (http://www.tactics-project.eu/).

### Compulsivity and compulsivity-associated disorders

Compulsivity and the closely associated impulsivity trait are characterised by behavioural disinhibition maintained by maladaptive fronto-striatal brain circuits [5]. Current neuroanatomical models posit the existence of separate but intercommunicating compulsive and impulsive fronto-striatal circuits, differentially modulated by neurotransmitters [5]. In the compulsive circuit, a striatal component (caudate nucleus) may drive compulsive behaviours and a prefrontal component (orbitofrontal cortex, OFC) may exert inhibitory control over them. Increased activity within the striatal components or decreased activity in the prefrontal components may thus result in an increased automatic tendency for executing impulsive or compulsive behaviours, depending on the sub-component affected. Childhood OCD has recently been associated with decreased prefrontal activity [6], fitting with reduced prefrontal control over the striatum. Interestingly, glutamatergic imbalance in fronto-striatal regions has also been observed in childhood OCD [7]. Furthermore, earlier work has shown reduced volume of the striatum in children with ASD that relates to repetitive and compulsive behaviours [8, 9].

### Glutamatergic mechanisms and fronto-striatal circuits

The fronto-striatal circuits implicated in compulsivity and impulsivity are notable for their relatively rich glutamatergic receptor density [10]. Moreover, glutamate modulates the metabolism and function of the fronto-striatal circuits. Glutamatergic projections to and from the various frontal sub-regions (OFC, infralimbic cortex and prelimbic cortex) to the striatum play a key role in the regulation of various compulsive behaviours in humans including: (1) the feeling of loss of control (OCD), (2) repetitive motor behaviours (stereotypy in ASD), and (3) a maladaptive habitual pattern (addiction). Neuroimaging studies have confirmed that glutamatergic projections between the various frontal sub-regions and the striatum play a key role in the regulation of compulsive behaviours in humans ([10, 11]. Compulsivity has been associated with increased glutamatergic activity in the striatum and the anterior cingulate cortex in clinical magnetic resonance spectroscopy (MRS) studies [11].

### Glutamatergic medication in the treatment of compulsivity/impulsivity

Despite the effectiveness of serotonergic compounds in the treatment of OCD, treatment-resistant symptoms remaining in 40 to 60 % of patients present a pressing clinical problem [12–14]. Thus, there is an urgent need for the development of conceptually novel pharmacological strategies. Consistent with the hypothesis of the role of glutamatergic dysregulation in the pathophysiology of OCD, agents targeting glutamate neurotransmission, such as memantine (and riluzole), represent promising candidates (e.g. [13–17]). Open-label studies in children and adults have provided supportive evidence for the effectiveness of these glutamate-modulating compounds for OCD symptoms [12, 13, 18–22]. There are currently no drugs that have been shown to effectively treat the core impairments of ASD. Although studies of serotonin reuptake inhibitors and atypical antipsychotics have suggested improvement of repetitive behaviour in ASD, this has yet to be proven in placebo-controlled trials [23–25]. Emerging evidence from neuroimaging studies suggests: individuals with ASD show an imbalance of excitatory (glutamate) and inhibitory neurotransmitters (gamma-aminobutyric acid (GABA)). Such regionally specific abnormalities in glutamatergic neurotransmission may vary across the lifespan [26, 27]. Ghanizadeh reviewed investigations of amino acid levels in plasma, serum and cerebrospinal fluid (CSF) from ASD patients and reported both increased levels of glutamate and homocysteine and decreased levels of glutamine and tryptophan [28].

### Systematic literature review and choice of investigational compound

A systematic literature review (Mechler et al., in preparation) has recently been performed within the TACTICS network, in preparation for this exploratory ‘Glutamatergic medication in the treatment of Obsessive Compulsive Disorder and Autism Spectrum Disorder’ (GOAT) trial. It focused on the evaluation of glutamatergic substances with potential benefit for the treatment of paediatric patients in multi-centre studies aiming at compulsivity-related disorders. In summary, there were a total of 21 trials (paediatric randomised controlled trials and open-label trials). Six substances were evaluated: D-cycloserine, memantine, minocycline, modafinil, *N*-acetylcysteine, and riluzole. The most substantial information appeared to be available for the compound memantine. Memantine is an *N*-methyl-D-aspartate (NMDA) antagonist, clinically used as a ‘cognitive enhancer’, regulatory-approved for the treatment of Alzheimer’s dementia in a number of countries. It has successfully been used and further been studied in children diagnosed with ASD for the treatment of cognitive, behavioural, and memory dysfunctions, in open-label and controlled trials [29–31]; cf. a recent clinical development program (memantine) sponsored by Forest Laboratories/Merz Pharmaceuticals, www.clinicaltrials.gov). Memantine has also been explored for the treatment of attention deficit/hyperactivity disorder (ADHD) (e.g. [32]). However, additional research examining the efficacy/effectiveness and tolerability/safety of memantine, e.g. placebo-controlled clinical trials, is needed. Findings on memantine suggested clinical effectiveness in ASD and OCD symptomatology and appeared to offer the best risk-benefit profile for multi-centre trials with paediatric patients suffering from compulsivity-related disorders. Considering the current state of knowledge and assessing available findings on effectiveness, tolerability/safety and feasibility of study implementation, the subgroup of experts in paediatric psychopharmacology from the TACTICS consortium jointly decided to apply the compound memantine for this paediatric pilot study.

### Molecular-genetic factors involved in compulsivity, in ASD and OCD

Compulsivity-related disorders such as OCD and ASD are assumed to be highly heritable but genetically complex, with multiple genetic variants of small effect contributing to disease in most patients [33]. Genes encoding the SLC1A1, SLC1A2 and SLC1A3 glutamate transporters are candidate genes for both OCD and ASD [34]. Genes encoding the NMDAR1 [35] and NMDAR2A [36] subunits of the postsynaptic NMDA glutamate receptor are associated with ASD and those encoding the NMDAR2B subunit with paediatric OCD [37]. Novel approaches are needed to facilitate the identification of genetic risk factors for brain disorders and related traits, like compulsivity. One such approach is the study of intermediate phenotypes [38]. It has been shown that using intermediate phenotypes indeed did improve the chance of establishing significant findings in genetic studies [39–41].

### Biomarkers – proteomics of compulsivity and associated disorders

Symptoms of compulsivity-associated disorders such as OCD, ASD and ADHD frequently co-occur in the same patients [42]. Quantitative and molecular genetics, cognitive and imaging studies indicate that this clinical overlap is due to common genetic factors and/or common underlying neural and molecular mechanisms (review, see [43]). Indeed, it has been shown that alterations in brain development, mitochondrial function, immune system and oxidative stress pathways as well as dysfunctions in neurotransmitter systems may be involved in several or all of these disorders [44–47]. The biological determinants that contribute to the pathogenesis of these conditions, however, are poorly defined, and currently, no well-characterised biomarkers are available. One major positive impact of this study – as integral part of the TACTICS project – may lead to a greater insight into the molecular mechanisms involved in the development of compulsivity and compulsivity-associated disorders and potentially facilitate the development of much needed differential diagnostics, improved drug discovery and clinical development, and designs based on biomarker-based segmentation of patients. This may allow for earlier diagnosis and personalised medicine strategies for more effective treatments in the future [48–51].

### Aims and objectives

#### Primary objectives

To investigate: clinical effectiveness of the glutamatergic compound memantine in paediatric patients with OCD (GOAT-1) and ASD (GOAT-2) with respect to symptoms of ‘compulsivity’ (assessed as total score of the Children’s Yale-Brown Obsessive-Compulsive Scale (CY-BOCS; [52])) and to explore tolerability and safety (based on, e.g. laboratory measures, electrocardiography (ECG), adverse events) of the compound memantine in these clinical indications.

#### Secondary objectives

To explore: the effects of glutamatergic interventions at the level of structure, function and biochemistry of the fronto-striatal circuits (magnetic resonance imaging (MRI), MRS), in subgroups (e.g. disorders, age range).

To collect: blood for genetic analyses and biomarkers.

To explore: additional clinical outcomes, disorder-specific outcomes (e.g. core symptoms, response rates, social functioning).

#### Tertiary objectives

To explore: the role of new candidate genes and pathways for compulsivity by linking genes to clinical phenotypes, neurocognitive test performance, and key structural and functional neuroimaging measures of the fronto-striatal circuits.

To explore: biomarkers/proteomics for compulsivity traits.

## Methods/design

This study is a multi-national, multi-centre, randomised, double-blind, parallel, placebo-controlled add-on trial with three study periods: study period I will be a 2-week screening (and washout) period, study period II will be a 12-week double-blind, randomised, placebo-controlled period, and study period III will be a double-blind 1-week down-titration period. Study medication (memantine or placebo) will be added to regular treatment for the respective disorders. Male and female patients (*N* = 50 per disorder, i.e. OCD, ASD), will be randomised into study period II. Patients who complete study period II will participate in study period III. The study visit intervals (suggested and allowed) for the three study periods are outlined in detail as part of the schedule of events (SOE) (Table 1).

**Table 1:** Schedule of events

Study period (SP)	SP I (screening/ washout)		SP II (double-blind treatment)						SP III (down-titration)			
Visit number:	1	2	3	4	5	6	7	8	9	10	Unscheduled visits^a^	Early discontinuation
(End of) week:	−2	−1	0	1	2	4	6	8	12	13		
Suggested time to next visit (days):	7	7	7	7	14	14	14	28	7			
Allowable time to next visit (days):	5–9	5–9	5–9	5–9	10 - 18	10–18	10–18	24–32	5–9			
Clinical assessments:												
Informed consent/assent	X											
Psychiatric assessment	X											
Systematic Interview, e.g. DISC (OCD group)	X											
Systematic Interview, e.g. DISC (re co-morbidities): ODD, CD, tic domains (all patients)	X											
ADI-R short version, (ASD group)	X											
WIS-C (4 subtests)	X											
Inclusion/exclusion criteria	X	X	X									
Randomisation to active drug vs. placebo			X									
Demographics	X											
Physical examination	X											
Medical history	X											
Discontinuation of prohibited medications	X											
Height, weight, vital signs	X								X			X
Clinical laboratory tests^b,c,^	X		X^c^			X			X^c^		(X)^a^	X^c^
Serum and urine pregnancy test^d^	X											
Urine drug screen	X										(X)^a^	X
Urine analysis	X		X			X			X			X
Biomarkers			X									
Genetics			X									
Physical Development Scale			X						X			
CGI-S	X		X	X	X	X	X	X	X	X		X
CGI-I				X	X	X	X	X	X	X		X
C-GAS			X			X			X			X
CBCL, 6–18			X									
CY-BOCS			X	X	X	X	X	X	X	X	(X)^a^	X
Children’s Social Behaviour Questionnaire			X	X	X	X	X	X	X	X	(X)^a^	X
Repetitive Behaviour Scale-Revised (RBS-R),			X	X	X	X	X	X	X	X	(X)^a^	X
CHIP-CE/AE			X			X			X			X
PAERS			X	X	X	X	X	X	X	X	X	X
Neuropsychological test battery			X						X			X
Neuroimaging assessments			X						X			
AE monitoring		X	X	X	X	X	X	X	X	X	X	X
Concomitant medications	X	X	X	X	X	X	X	X	X	X	X	X
Eligibility to remain in study		X	X	X	X	X	X	X	X	X	X	X
Study drug dispensed			X	X	X	X	X	X	X			
Study drug returned				X	X	X		X	X	X		X

^a^laboratory tests, ECG, etc. at unscheduled visits (due to AEs) according to the discretion of the investigator

^b^patients with significant increase after baseline (visit 3) in AST, ALT, total bilirubin, or AP from the upper limit of the lab reference range will have additional lab testing and/or consultation

^c^to be collected in a fasting state at visits 3 and 9 (and early discontinuation, if possible); fasting defined as at least 8 hours with water as the only oral consumption

^d^females of child-bearing potential only; additional pregnancy tests possible at each visit at the discretion of the investigator

*AE* adverse event, *ASD* autism spectrum disorders, *AST* aspartate aminotransferase, *ALT* alanine aminotransferase, *AP* alkaline phosphatase, *CBCL* Child Behaviour Checklist, C-GAS Children’s Global Assessment, CGI-I, Clinical Global Impression-Improvement, CGI-S Clinical Global Impression-Severity, *CHIP-CE/AE* Child Health and Illness Profile, *CY-BOCS* Children’s Yale-Brown Obsessive-Compulsive Scale, *DISC* Diagnostic Interview for Children, *ECG* electrocardiography, *OCD* obsessive-compulsive disorder, *PAERS* Pediatric Adverse Event Rating Scale

### Participating sites

Participating sites are Mannheim (Department of Child and Adolescent Psychiatry and Psychotherapy, Central Institute of Mental Health), Germany; London (Departments of Neuroimaging and Child and Adolescent Psychiatry, Institute of Psychiatry, Psychology and Neuroscience, King’s College London), United Kingdom; Nijmegen (Karakter Child and Adolescent Psychiatry, Donders Institute for Brain, Cognition and Behaviour, Radboud University Medical Center), The Netherlands and Utrecht (Department of Child and Adolescent Psychiatry, Brain Center Rudolf, University Medical Center Utrecht), The Netherlands.

### Information and consent procedure

Subjects and their parents will be asked to participate in this study by one of the (sub-) investigators. They will receive a letter/informed consent document explaining the study (in their local language). All children and adolescents will also receive a respective letter/informed assent document about the study, with the (complexity of the) language suitably adapted. After an adequate time interval, the subjects’ parents will be contacted again. If subjects require more time to consider participating, as much time as is necessary will be allowed. Informed consent and assent will be obtained from all guardians and participants before inclusion. If the patients and parents decide to participate, a brief screening will be conducted, e.g. on the telephone, to check for any major exclusion criteria. If after this brief screening the subject is included in the study, they will receive a set of questionnaires at home. Parents will be asked to fill out these forms and return these to the centre.

### Study period I (screening period with washout)

Study period I is a 2-week screening and washout period (visits 1–2); during this period, patients will be screened for study eligibility. Before or at the day of visit 1, the study will be explained to the patient and his or her parent or legal guardian, who will then sign and date the informed consent and assent documents (*International Classification of Diseases* (ICD); as adequate according to the legal requirements in the respective country). The ICD must be signed before any study procedures are conducted and before patients discontinue any excluded medications.

Screening for eligibility begins at visit 1. If the patient is eligible, the patient will enter the washout phase during which all excluded medications will be tapered off and stopped entirely by visit 2 (for the respective compounds, the medication-free period should be at least five half-lives in duration). Randomisation to active drug or placebo will occur at visit 3. At visit 1, patients will undergo psychiatric screening tests and safety screening procedures. A patient history will also be taken. See the study SOE (Table 1) for a complete list of study period I procedures. All criteria for enrolment, including the ECG and laboratory analyses, will be verified prior to randomisation at visit 3. At visits 2 and 3, patients will be further evaluated by the clinical research team as shown in the study SOE (Table 1).

### Study period II (acute period)

Study period II is a 12-week, randomised, double-blind, and placebo-controlled add-on acute treatment period (visits 3–9). At baseline (BL), patients will randomly be assigned to active drug or placebo in a 1:1 ratio. In order to safeguard patients in the study, patients can be either in outpatient or inpatient status. For patients in both the active drug treatment group and in the placebo group, dosage will be given depending on the patient’s weight, and will be up-titrated according to Table 2. Visit 7 will be performed via a telephone interview with assessments of effectiveness and tolerability/safety (no physical examination, ECG, blood draws, etc.); if needed from a clinical perspective ‘unscheduled visits’ will be performed, based on investigator judgment.

**Table 2:** Memantine dosing schedule in mg/day by body weight; study period II

	Dose range (mg/day)^a^ of distributed medication at … (for weeks …):					
Screening/Baseline Weight (kg)	V3 (wk 1)	V4 (wk 2)	V5 (wks 3–4)	V6 (wks 5–8)	V8 (wks 9–12) ^b^	V9 (wk 13) (discontinuation)
≥60	5	10	15	15	15	10 → 5 → 0
40–59	5	10	10	10	10	5 → 0
20–39	5	5	5	5	5	5 → 0

Dose titration aims at target doses as indicated in the table, based on clinical judgment.

^a^Dosage may be adjusted upwards and downwards by 5 mg/day increments within the range shown for each weight group at indicated visits/dates

^b^Dose should remain stable during the last 4 weeks of study period II (weeks 9–12), unless a dose reduction is necessary for tolerability or safety reasons, based on clinical judgment

### Study period III (double-blind down-titration period)

Study period III is a 1-week double-blind down-titration period (visits 9–10) from study medication, active drug or placebo. Any patient who completes study period II (visit 9) will continue into study period III. In a blinded fashion, their dose of study medication will be titrated down depending on their final dose in study period II. Placebo-treated patients from study period II will continue placebo treatment in study period III. In order to maintain the blind, all patients will receive the respective amounts (e.g. number of tablets) of study drug (active or placebo). The final visit of this period will be visit 10. See the SOE for the timing of events and the measures to be assessed (Table 1).

### Study population

Overall, *N* = 100 patients (*N* = 50 participants with OCD and *N* = 50 participants with ASD), male or female, with inpatient or outpatient status, aged 6 years (ASD patients)/8 years (OCD patients) to 17 years and 9 months at inclusion, will be randomised into study period II. These patients will be recruited from four centres, i.e. per site approx. *N* ≥24 children and adolescents (*N* = 12–13 with OCD; 12–13 with ASD).

### Inclusion criteria

General inclusion criteria for both patient groups (OCD and ASD):

- Male or female patients
- Inpatient or outpatient status
- Aged 6 years (ASD patients)/8 years (OCD patients) to 17 years 9 months at initial inclusion
- IQ ≥70 (based on Wechsler scales, four subtests)
- Clinical Global Impressions-Severity (CGI-S; [53, 54]) score ≥4 (moderately ill; anchored to respective disorder) at BL (visit 3)
- Ability to speak and comprehend the native language of the country in which the assessments take place
- Signed informed consent by parents or legal representatives
- Signed informed assent by patients (indicating that the subject is aware of the investigational nature and the core aspects of the study and the study is run in accordance with the International Conference on Harmonisation of Technical Requirements for Registration of Pharmaceuticals for Human Use (ICH) Good Clinical Practice (GCP) guideline E6 (1996)
- Female subject must not have a positive beta-HCG pregnancy test at screening, and must have a negative urine pregnancy test at BL, and agree to comply with applicable contraceptive requirements

Inclusion criteria for subjects with OCD (GOAT-1):

- *Diagnostic and Statistical Manual, 5th edition* (DSM-5) diagnosis of OCD [2], according to a structured interview (e.g. DISC; [55])

Inclusion criteria for subjects with ASD (GOAT-2):

- DSM-5 diagnosis of ASD [2], according to the Autism Diagnostic Interview-Revised (ADI-R; [56])

### Exclusion criteria

General exclusion criteria for all subject groups:

- Intellectual disability (IQ <70)
- Body weight below 20 kg at BL
- Major physical illness of the cardiovascular, endocrine, pulmonary, or the gastrointestinal system
- Contra-indications for memantine, according to the Summary of Product Characteristics (SPC)
- History of or present clinically relevant somatic acute or chronic disorder that, in the opinion of the investigator, might confound the results of tolerability/safety assessment, or prohibit the patients from completing the study, or would not be in the best interest of the patient
- Subject has failed to respond, based on investigator judgment, to an earlier adequate course (dose and duration) of the investigational drug therapy, memantine
- Subject has a documented allergy, hypersensitivity, or intolerance to, memantine
- Subject has a positive urine drug screen result at screening or BL (apart from earlier prescribed medication; retest and negative result at BL needed then)
- Subject has taken another investigational product or taken part in a clinical study with 30 days prior to screening
- Subject is female and is pregnant or lactating
- For those subjects intending to participate in the neuroimaging assessments: all contra indications for MRI assessment, such as the presence of metal objects in or around the body (e.g. pacemaker, dental braces)
- Patients/parents will not be excluded from the study if they do not agree to participate in certain sets of assessments (e.g. neuroimaging, blood sampling for bioanalytical analyses)

### Randomisation

Randomisation will be done in a 1:1 ratio (active drug: placebo).

### Sample size calculation

The total sample size of approx. *N* = 100 paediatric patients (*N* = 50 in each diagnostic subgroup (OCD, ASD)) across trials will not allow for confirmatory statistical analyses but will be adequate for descriptive statistics, for pilot-testing of the chosen glutamatergic medication (memantine), and for hypothesis generation.

### Endpoints

The endpoint for primary objective 1 (clinical effectiveness) is the baseline-to-endpoint change of compulsivity as measured by the CY-BOCS total score (all patients). The endpoints for primary objective 2 (tolerability and safety) are adverse event (AE) rates, derived from both spontaneous reporting and a structured AE instrument, the (adapted) Pediatric Adverse Event Rating Scale (PAERS; [57]).

The endpoints for the secondary objectives (other effects) are additional clinical outcomes, e.g. response rates, response defined as at least 30 % reduction versus BL on the primary outcome scale (CY-BOCS total score, for OCD, ASD patients) plus Clinical Global Impressions-Improvement (CGI-I; [53, 54]) score of 1 (very much improved) or 2 (much improved); baseline-to-endpoint changes in the total score of the Aberrant Behaviour Checklist-Community (ABC-C; [58–60]: ASD patients).

For the tertiary objective (genetic pathways) genotypes of single common and rare variants in candidate genes, and also combined genetic variants in whole genes or neurotransmitter systems/gene pathways will be investigated and further laboratory assessments of various proteins in blood plasma will be performed. Neuroimaging data will be analysed according to the TACTICS COMPULS protocol (see below) in order to explore the effects of glutamatergic interventions at the level of the structure, function and biochemistry of the fronto-striatal circuits.

### Measures

#### Diagnostic instruments

##### Diagnostic Interview for Children (DISC)

In order to determine diagnoses of OCD, e.g. the Diagnostic Interview for Children (DISC; [55]) will be used and administered by trained investigators. In order to assess most common co-morbidities, the modules for tic disorder, oppositional-defiant disorder, and conduct disorder will be applied, additionally; this interview will be completed with parents of patients in all groups.

##### Autism Diagnostic Interview-Revised (ADI-R)

The Autism Diagnostic Interview-Revised (ADI-R; [56]: adapted short version, focusing on criteria relevant for diagnosis) will be administered by a certified interviewer to determine the ASD diagnosis. This interview covers the core domains of ASD.

##### Clinical change

Rigid and compulsive patterns of behaviour will be measured in all participants, thus both patient groups, using the following measures:

- *Aberrant Behaviour Checklist-Community (ABC-C)*
  The Aberrant Behaviour Checklist-Community (ABC-C; [58, 59]) is a parent-rated 58-item scale assessing behaviour and psychiatric symptoms across the five domains: Irritability, Agitation and Crying, Lethargy/Social withdrawal, Stereotypic behaviour, Hyperactivity/Noncompliance and Inappropriate speech.
- *Children’s Yale-Brown Obsessive-Compulsive Scale (CY-BOCS)*
  The Children’s Yale-Brown Obsessive-Compulsive Scale (CY-BOCS; [52]) is a clinician-rated 10-item scale, with each item rated on a 5-point scale from 0 = ‘no symptoms’ to 4 = ‘extreme symptoms’, with, apart from a total score, subtotals for obsessions (items 1–5) and compulsions (items 6–10).
- *Children’s Social Behaviour Questionnaire (CSBQ)*
  The Children’s Social Behaviour Questionnaire (CSBQ; [61–63]) is a 49-item parent-rated questionnaire that aims to quantify the different behavioural dimensions along which children with ASD vary. In this study we will use the total score and rigidity(-related) subscale scores (e.g. Stereotyped behaviour, and Fear of and resistance to changes).
- *Repetitive Behaviour Scale-Revised (RBS-R)*
  The Repetitive Behaviour Scale-Revised (RBS-R; [64]) is a 43-item parent-rated questionnaire for measuring the presence and severity of a variety of forms of restricted, repetitive behaviour. It consists of six subscales: Stereotyped behaviour, Self-injurious behaviour, Compulsive behaviour, Routine behaviour, Sameness behaviour, and Restricted behaviour.
- *Clinical Global Impression-Severity (CGI-S)*
  The Clinical Global Impressions-Severity (CGI-S; [53, 54]) is a single-item rating of the clinician’s assessment of the severity of symptoms in relation to the clinician’s total experience with OCD or ASD patients. Severity is rated on a 7-point scale (1 = normal, not at all ill; 7 = among the most extremely ill subjects).
- *Clinical Global Impression-Improvement (CGI-I)*
  The Clinical Global Impressions-Improvement (CGI-I) [53, 54] is a single-item clinician rating of the total improvement (or worsening) of the patient’s symptoms since the beginning of treatment regardless of whether the improvement (or worsening) was thought to be due entirely to the drug treatment. Improvement/worsening is rated on a 7-point scale (1 = very much improved; 7 = very much worsened).
- *Children’s Global Assessment (C-GAS)*
  The Children’s Global Assessment Scale (C-GAS; [65]) is a global, unidimensional clinician rating of social and psychiatric functioning in children aged 4–16 years. Scores on the measure range from 1 (most impaired) to 100 (healthiest).

#### Additional measures

##### The Child Behaviour Checklist (CBCL, 6–18)

The Child Behaviour Checklist (CBCL, 6–18; [66] cf. [67]) will be applied – parent-reported – at BL in order to assess general symptomatology (cf. SOE). It is a standard instrument that allows evaluating various behavioural domains in children and adolescents; it has been psychometrically validated and is available in the very country languages involved in this study. In this study, the ‘problem behaviour’ section will be applied only, not the ‘competencies’ part. It may also allow for analyses on mediating factors for primary and secondary outcome parameters.

#### Health outcomes/Quality of life measures

##### Child Health and Illness Profile (CHIP-CE)

The Child Health and Illness Profile (CHIP-CE) Parent Report Form [68] has been used in a number of paediatric trials with psychotropic medications and allows comparisons with findings from paediatric populations with non-psychiatric disorders. It is available in the respective country languages involved in this study.

#### Safety evaluations

##### Spontaneous adverse event reporting

Spontaneous adverse event reporting will be based on standard questioning of patients and parents by the investigator at each visit.

##### Pediatric Adverse Event Rating Scale (PAERS)

In a second step, the structured Pediatric Adverse Event Rating Scale (PAERS; [57]: adapted/shortened version) will be applied at each visit, beyond standard AE reporting solicited by open questioning, in order to systematically collect AEs; the investigator-rated version will be used, based on parent- and patient-information.

Only PAERS reports on ‘serious’ AEs will be added to the ‘spontaneous reporting of AE results’ and be reported immediately.

### Laboratory tests

Blood and urine samples will be collected at the times specified in the study SOE (Table 1) and when clinically indicated. Standard laboratory tests including clinical chemistry, electrolytes, urine analysis, and haematology panels will be performed. Laboratory tests will be analysed by a local laboratory, at each study site. Urine drug screening will be performed at study start and in case of suspected intoxication, overdose, etc., at the discretion of the investigator, at regular or unscheduled visits. If agreed to by patients and parents, 30 ml blood will be drawn from participants for biobanking purposes, for DNA isolation, and assessment of biomarkers for which either serum or plasma samples are prepared. The venipuncture will be performed by medical staff, e.g. a trained nurse practitioner, who has considerable experience of working with children.

### Molecular-genetic material

Genome-wide genotyping of common and more rare variants will be performed using PsychChip (or similar) (https://sites.google.com/a/broadinstitute.org/psych-chip-resources/home). The resulting data will be analysed in two ways: (1) the variation in the genes will be analysed for rare variants that appear to have deleterious effect on gene function (based on conservation, predicted changes in gene-product structure and function). Those will be studied further by protein studies; and (2) in addition to this, gene-wide burden analyses [69] of rare as well as common variants will be performed to identify an association with treatment response or diagnosis. All analyses will be corrected for multiple testing as appropriate.

### Other biological material

Protein levels of blood-expressed compulsivity and addiction genes will be monitored using multiplex immunoassay profiling of peripheral blood cells and serum. This work will be performed in close collaboration with the laboratory of Professor S. Bahn in Cambridge/UK. In addition, at the Nijmegen centre there are expertise and facilities for High Pressure Liquid Chromatography (HPLC) in blood platelets (for products of neurotransmitter pathway genes). Earlier work has shown that potential compulsivity- and addiction-relevant molecules such as hormones, cytokines and neurotransmitters such as glutamate and serotonin, are measurable in serum and blood cells.

### Neuropsychological assessment

All neuropsychological tasks performed within this study are suitable for subjects from the age of 5 years to adulthood.

#### Neurocognitive assessment battery

In order to explore potential medication effects (improvements, impairments) to cognitive functioning, a neurocognitive assessment battery, the Amsterdam Neuropsychological Test Battery (ANT), will also be used, including attentional, set-shifting, motor speed and inhibition, cognitive flexibility, and verbal working memory tasks.

#### Wechsler Intelligence Scale for Children (WISC-III)

The WISC-III will be performed in order to determine the general IQ level of the patient if not done within 2 years from BL [70]. Patients must have an IQ of >70 (based on, at least four subtests, two verbal plus two performance tests) from the Wechsler IQ Scales, e.g. WISC, WAIS. In this study four subtests will be used, e.g. Vocabulary, Similarities, Block Design, and Matrix reasoning [71]. Age- and country-specific adaptations may be used.

### Neuroimaging

If agreed to by patients and parents, neuroimaging of the patients will be performed by MRI according to the protocol of another TACTICS project, COMPULS. The aim of COMPULS is to identify neural, neurocognitive, genetic, and biomarker mechanisms underlying compulsive behaviours in high-risk subjects and controls. The primary objectives are to examine whether (1) structural and functional abnormalities of the fronto-striatal circuits are related to the development of compulsive and impulsive behaviour in ASD and OCD, and (2) the glutamate system is a key modulator in these fronto-striatal circuits. The COMPULS study has a mixed cross-sectional longitudinal design with assessments at two time points. Measurements are: MRI (structural MRI, functional MRI, resting state MRI, Diffusion Tensor Imaging (DTI)), MRS (spectroscopy), plus multiple tasks reflecting cognition and behaviour (cf. website: http://www.tactics-project.eu; WP04).

### Vital signs

Vital signs including body temperature, heart rate (pulse), and blood pressure will be recorded as specified in the study SOE (Table 1).

### Height and weight

Height and weight will be recorded as specified in the study SOE (Table 1); measurements will be performed according to standardised procedures across study sites.

### Electrocardiogram (ECG)

ECGs will be performed following standard procedures at the visits outlined in the SOE (Table 1).

### Medication and dosing

The investigator or his/her designee is responsible for explaining the correct use of the investigational agent(s) to the patients and parents, verifying that instructions are followed properly, maintaining accurate records of study drug dispensing and collection, and of returning all unused medication at the end of the study.

This study involves a comparison of an active drug (memantine) with matching placebo. During study period II, medication will be initiated and modified according to the weight group of the patient at BL (visit 3). Dosing may then be increased (or decreased) by 5 mg/day increments, respectively, at specified visits/dates according to the weight of the subject (cf. Table 2). Stepwise up-titration is recommended to the target dose or the highest tolerable dose by week 4, accordingly. Dosing should remain stable during the last 4 weeks of study period II unless a dose reduction is necessary for tolerability or safety reasons. In case of dose-adjustments due to AEs unscheduled visits can be performed (for stepwise down-titration). Patients who cannot tolerate the minimum daily dose specified (5 mg/day) will be discontinued from the study. Investigator decisions on dose changes (up or down) will only be permitted at study visits (or, due to AEs, at unscheduled visits for down-titration). Study medication will be given once daily in the evening, with or without food.

Based on the dosing information from the published literature and considering earlier and ongoing trials with memantine (sponsored by the pharmaceutical company Forest Laboratories, Berlin, Germany) in this age group, dosing was chosen weight-based, accordingly, also taking into account dose-unit availability in Europe (see Table 2).

### Production, packaging of the medication

All study medication will be identical in appearance and will be dispensed to patients or their parents by trained site personnel. A cooperation partner (H. Lundbeck A/S Pharmaceuticals, Copenhagen, Denmark) will provide the primary study material consisting of tablets containing 5 and 10 mg of memantine each and tablets containing placebo (in identical appearance). Study medication will be dispensed to the patient/parent at the investigator’s study site. Study medications will be packaged in blister packs or bottles. Clinical trial materials will be labelled according to the country’s regulatory requirements.

### Concomitant therapy

In general, concomitant medications with primarily central nervous system activity are not permitted in this study. A protocol attachment contains a list of medications and classes of medications that are or are not permitted during this study (depending and in accordance with the SPC for memantine, used in GOAT-1 and GOAT-2). Upon assent/consent by the patient and patient’s parent, the medications that are not allowed should be tapered off and stopped by visit 2, and be completely washed out by visit 3. Investigators should follow clinical guidelines with respect to duration of washout periods (minimum of five half-lives). Concomitant medication will be reported with compound, length of use, and doses in the clinical report form.

### Treatment compliance

Patient compliance with study medication will be assessed at each visit by direct questioning of parent and/or patient. Patients/parents will be asked to return both used (including empty) and unused blister cards distributed on the previous visit. Any deviation from the prescribed dosage regimen will be recorded. Final drug accountability reconciliation will be performed at the end of treatment or discontinuation.

During the double-blind treatment phase (study period II), a patient will be considered significantly noncompliant if he or she misses more than two consecutive days of study medication (full doses), or fails to take at least 80 % of prescribed doses of study medication (full doses) during two or more visit intervals. Similarly, a patient will be considered significantly noncompliant if he or she intentionally or repeatedly takes more than the prescribed amount of medication as judged by the investigator.

If a patient is noncompliant during an interval, the patient and parent will be counselled regarding the importance of compliance in this study.

### Statistical analysis

#### Clinical efficacy data

The end of the treatment phase will be taken as the primary endpoint. Change from BL in CY-BOCS total score will be used as the primary efficacy variable. As a secondary outcome measure a binary response score will be composed based on the change in CY-BOCS total score and the Clinical Global Impression-Improvement (CGI-I) scale at the end of the treatment phase (at least 30 % reduction in CY-BOCS total from BL and a score of 1 (very much improved) or 2 (much improved) in CGI-I scale). Additional symptom severity scores will be used as secondary efficacy measures.

General linear models will be applied on the CY-BOCS change score to compare the two treatment groups (active drug, placebo). Gender, age, investigational site and BL measurement will be included as covariates. Furthermore, diagnosis and diagnosis by treatment interaction will be entered in the model. A significant interaction effect will be followed up by separate analyses of the two diagnostic groups. In the presence of a high drop-out rate methods for missing data (e.g. last observation carried forwards (LOCF), mixed effect model repeat measurement (MMRM)) will be applied and sensitivity analyses will be performed to assess whether efficacy results are stable across different analysis methods. Logistic regression analyses will be performed to evaluate the effect of treatment on the binary response score. To study the impact of moderating factors on the outcome measures general linear models and logistic regression analyses will be applied, as appropriate. Gender, age, diagnostic group and investigational site will be included as covariates in all analyses. Multivariate statistical analysis of all data will be applied, reducing the chances of identifying false positives. This method can incorporate different types of predictors (clinical patient characteristics as well as neuropsychological and neuroimaging data, genetic and protein data). All analyses will be corrected for multiple testing as appropriate.

#### Tolerability/Safety data

To analyse tolerability and safety of the medication logistic regression analysis will be performed on the indicator variables for AEs, and Poisson regression will be used for the number of AEs. Gender, age, diagnostic group and investigational site will be included as covariates in all analyses.

### Safety reporting

Investigators are responsible for monitoring the safety of patients who have entered this study and for alerting the representative of the sponsor to any event that seems unusual. The investigator is responsible for appropriate medical care of patients during the study.

### Reporting to study participants (parents and patients)

The sponsor (Radboud University Medical Centre, Nijmegen, Prof. Dr. J. K. Buitelaar) or the respective representative at each study centre will take responsibility to report serious adverse events (SAEs) to the regulatory bodies and Ethical Review Boards (ERBs) in the countries as well as to investigators involved in this study, if applicable in close collaboration with the contract research organisation involved in this study.

### Adverse and serious adverse events

AEs are defined as any undesirable experience occurring to a subject during the study, whether or not considered related to the study. ‘A serious adverse event (experience) or reaction is any untoward medical occurrence that at any dose: results in death, is life-threatening, results in persistent or significant disability/incapacity, or is a congenital anomaly/birth defect, or requires inpatient hospitalisation or prolongation of existing hospitalisation’ [72]. The participating study centres comply with national guidelines for procedures in the case of (S)AEs, and these rules will be followed in case an AE occurs. All AEs occurring during the visit of the participant to the test site, or reported spontaneously by the patient or parent, or observed by the investigator or his staff will be recorded. All AEs will be followed until they have abated, or until a stable situation has been reached. Depending on the event, follow-up may require additional tests or medical procedures as indicated, and/or referral to a general physician or medical specialist.

### Withdrawal of individual subjects

Patients can leave the study at any time for any reason if they or their parents wish to do so without any consequences. The investigator can decide to withdraw a subject from the study for urgent medical reasons. Study participation will also be cancelled if patients do not comply with the general directives of the study procedures, or if the investigator decides it is in the best interest of the subject (medical-ethical considerations).

The participating centres have considerable experience with the type of research in paediatric populations proposed in this protocol, and there are limited risks associated with this kind of research.

### Premature termination of the project

If the project is terminated prematurely, for whatever reason, the accredited/responsible ERB(s) and/or regulatory agencies will be notified immediately. The investigator will inform the patients and parents plus the reviewing accredited/responsible ERB and/or regulatory agencies if anything occurs, on the basis of which it appears that the disadvantages of participation may be significantly greater than was foreseen in the research proposal. The study will be suspended pending further review by the accredited ERB/regulatory agencies, except insofar as suspension would jeopardise the patients’ health. The investigator will take care that all patients/parents are kept adequately informed.

### Ethical considerations

This study will be conducted according to the principles of the Declaration of Helsinki, version of 2008, and in accordance with the Medical Research Involving Human Subjects Act (WMO, as well as according to the ICH GCP Guideline E6 (1996), EU Directive 2001/20/EC and applicable regulatory requirements and guidelines in the participating countries/regions.

The study received ethical approval from the ethical committee in Mannheim, Germany (Medizinische Ethikkommission II, Medizinische Fakultät Mannheim der Universität Heidelberg, Germany) on 15 January 2015. Mannheim represents the leading and coordinating site. Further applications for ethical approvals were made in the UK (National Research Ethics Service Committee London – Camberwell St Giles) and in the Netherlands (CMO (Commissie Mensgebonden Onderzoek) Regio Arnhem-Nijmegen). Recruitment in any centre will not begin until local ethical approvals have been obtained in addition to the ethical approval of the leading site in Mannheim.

### Benefits and risks assessment, group relatedness

The risks and discomforts of this study are estimated as low. Any AE will be observed and addressed (e.g. by dose adjustment or early discontinuation from the study if clinically indicated).

### Handling and storage of data and documents

All study data will be handled confidentially. Each participant will get a code number which will be used to store all data linked to participation in the study and unlinked to demographic data (name, address, etc). The list linking the code numbers to the demographic data will be stored by the principal investigator. All data from all sites will be stored in a secured web-based database. Original data of all source documents will be retained for a period of at least 15 years after report of the study has been finalised. After analysis, any leftover material of biological nature will be stored at −20 °C or −80 °C (as appropriate) at the analysis location until 25 years after the study is finished. The key to the identity of participants will only be available to the principal investigators.

### End of study report

The investigator will notify the accredited ERB of the end of the study within a period of 8 weeks. The end of the study is defined as the last patient’s last visit. In case the study is ended prematurely, the investigator will notify the accredited ERB, including the reasons for the premature termination.

Within 1 year after the end of the study, the investigator/sponsor will submit a final study report with the results of the study, including any publications/abstracts of the study, to the accredited ERB.

### Public disclosure and publication policy

Findings from the present study are to be published in international, peer-reviewed scientific journals and presented at international conferences.

## Discussion

Based on the findings from the preclinical animal and human investigations within the TACTICS project, the planned proof-of-concept clinical study will allow pilot-testing of a (new) promising compound in children and adolescents with OCD or ASD. Drug treatment for children and adolescents with these indications has been a challenging issue because there have only been few placebo-controlled studies with glutamatergic compounds so far. In general, there is a lack of solid information on the effects of these drugs in children and adolescents; therefore, placebo-controlled studies will contribute to determine the tolerability/safety and efficacy/effectiveness in this population.

Due to a very limited budget as one of multiple projects within the TACTICS study, several cost-saving measures and solutions had to be implemented in the GOAT study design reflecting the pilot character of this study but not limiting scientific quality or jeopardising patients’ safety. This challenge has also been previously noted in other projects funded by the European Union, e.g. the Paediatric European Risperidone Studies (PERS) [73]. In TACTICS GOAT, e.g. interpretations of ECG and blood sample analyses as well as site monitoring will be performed locally at each study centre by qualified specialists rather than by a centralised system or clinical research organisation, respectively. This will be coordinated centrally by the leading study centre and performed in accordance with Standard Operating Procedures (SOP) for all sites. Furthermore, the pharmaceutical company H. Lundbeck A/S, Copenhagen, Denmark, became a member of the TACTICS consortium and will provide the medication free of charge. Cooperation between academia and pharmaceutical companies is one of the overarching objectives of European Union-funded projects. For packing, blinding, drug accountability and labelling we were able to find a collaborator in the Clinical Trials Unit of the Pharmacy at the University Hospital Heidelberg which supports investigator-initiated and publicly funded trials in a cost-saving yet professional manner.

Patients treated with glutamatergic compounds and their parents will have the benefit of potential medication effects on their symptomatology and the related burden of illness (reductions), plus of respective improvements in health-related quality of life. Equally important, all patients and parents can benefit from the high frequency and intensity of care – offered via the study visits by experienced child and adolescent psychiatry staff – that in general clearly extends beyond routine care settings.

An acute treatment period of as long as 12 weeks was chosen in order to increase the chance to reflect change/improvement based on medication treatment. With applying the respective adequate instruments, clinical outcome parameters will reflect compulsivity symptom clusters, but also comprehensive information on psychosocial functioning and health-related quality of life relevant in child and adolescent development. Contrasting findings across the patient populations will allow the assessment of disorder-related variation.

It is believed that the proposed project – with performing this pilot clinical trial in populations of children and adolescents with compulsivity disorders – will help to elucidate efficacy/effectiveness and tolerability/safety of glutamatergic medications, further to explore effects of moderating and mediating factors on the response to these compounds. Also, findings will allow assessment of medication effects on biomarkers and neural mechanisms underlying compulsivity. In addition, these pilot data will be made available to be used for potential future Paediatric Use Marketing Authorisation (PUMA) directed trial applications.

Respective MRI/MRS findings (from subgroups of patients, combined with the data from the TACTICS project’s COMPULS study) will help to clarify which mechanisms count for the compulsive behaviours in the considered disorders and subsequently to understand the links between compulsivity, impulsivity and addiction. These understandings will provide a basis for prospectively developing a guide for clinicians as to whether children are at high risk for negative outcome as severe compulsive behaviours and addictive behaviours in adolescence and adulthood.

## Trial status

At the time of manuscript submission, ERB approval for the leading site at the Central Institute of Mental Health in Mannheim, Germany (15 January 2015) plus German regulatory agency approval (BfArM, 26 June 2015) have been obtained. Recruitment of first patients into the trial is planned to begin in the second quarter of 2016.

This article is based on protocol version .01, 31 July 2014.

## Abbreviations

ADHD: attention deficit/hyperactivity disorder
ADI-R: Autism Diagnostic Interview-Revised
APA: American Psychiatric Association
ASD: autism spectrum disorder
CBCL: Child Behaviour Checklist
C-GAS: Children’s Global Assessment
CGI-I: Clinical Global Impression-Improvement
CGI-S: Clinical Global Impression-Severity
CHIP-CE: Child Health and Illness Profile
CSBQ: Children’s Social Behaviour Questionnaire
CSF: cerebrospinal fluid
CY-BOCS: Children's Yale-Brown Obsessive-Compulsive Scale
DISC: Diagnostic Interview Schedule for Children
DSM-5: *Diagnostic Statistical Manual, 5th edition*
EC: European Community
ECG: electrocardiography
EMEA/EMA: European Medicines Agency
ERB: Ethical Review Board
EU: European Union
FP7: Seventh Framework Programme
GABA: gamma-aminobutyric acid
GCP: Good Clinical Practice
GOAT: Glutamatergic medication in the treatment of Obsessive Compulsive Disorder and Autism Spectrum Disorder
HCG: human chorionic gonadotropin
HPLC: High Pressure Liquid Chromatography
*ICD*: *International Classification of Diseases*
ICH: International Conference on Harmonisation of Technical Requirements for Registration of Pharmaceuticals for Human Use
MRS: magnetic resonance spectroscopy
OCD: obsessive compulsive disorder
OFC: orbitofrontal cortex
PAERS: Pediatric Adverse Event Rating Scale
RBS-R: Repetitive Behaviour Scale-Revised
SOE: schedule of events
SOP: Standard Operating Procedure
SPC: Summary of Product Characteristics
TACTICS: Translational Adolescent and Childhood Therapeutic Interventions in Compulsive Syndromes
WAIS: Wechsler Adult Intelligence Scale
WISC: Wechsler Intelligence Scale for Children (WISC-III)
WMO: Wet Medisch Onderzoek (Law of Medical Research)

## References

[1] Chamberlain SR, Fineberg NA, Blackwell AD, Robbins TW, Sahakian BJ (2006). Motor inhibition and cognitive flexibility in obsessive-compulsive disorder and trichotillomania. Am J Psychiatry.

[2] American Psychiatric Association (2013). Diagnostic and statistical manual of mental disorders: DSM-5.

[3] Swedo SE, Rapoport JL, Leonard H, Lenane M, Cheslow D (1989). Obsessive-compulsive disorder in children and adolescents. Clinical phenomenology of 70 consecutive cases. Arch Gen Psychiatry.

[4] Anholt GE, Aderka IM, van Balkom AJ, Smit JH, Schruers K, van der Wee NJ (2014). Age of onset in obsessive-compulsive disorder: admixture analysis with a large sample. Psychol Med.

[5] Fineberg NA, Potenza MN, Chamberlain SR, Berlin HA, Menzies L, Bechara A (2010). Probing compulsive and impulsive behaviors, from animal models to endophenotypes: a narrative review. Neuropsychopharmacology.

[6] Britton JC, Rauch SL, Rosso IM, Killgore WD, Price LM, Ragan J (2010). Cognitive inflexibility and frontal-cortical activation in pediatric obsessive-compulsive disorder. J Am Acad Child Adolesc Psychiatry.

[7] MacMaster FP, O’Neill J, Rosenberg DR (2008). Brain imaging in pediatric obsessive-compulsive disorder. J Am Acad Child Adolesc Psychiatry.

[8] Langen M, Durston S, Staal WG, Palmen SJ, van Engeland H (2007). Caudate nucleus is enlarged in high-functioning medication-naive subjects with autism. Biol Psychiatry.

[9] Langen M, Schnack HG, Nederveen H, Bos D, Lahuis BE, de Jonge MV (2009). Changes in the developmental trajectories of striatum in autism. Biol Psychiatry.

[10] Monaghan DT, Yao D, Cotman CW (1985). L-[3H]glutamate binds to kainate-, NMDA- and AMPA-sensitive binding sites: an autoradiographic analysis. Brain Res.

[11] Rosenberg DR, MacMaster FP, Keshavan MS, Fitzgerald KD, Stewart CM, Moore GJ (2000). Decrease in caudate glutamatergic concentrations in pediatric obsessive-compulsive disorder patients taking paroxetine. J Am Acad Child Adolesc Psychiatry.

[12] Grant JE, Odlaug BL, Kim SW (2007). Lamotrigine treatment of pathologic skin picking: an open-label study. J Clin Psychiatry.

[13] Aboujaoude E, Barry JJ, Gamel N (2009). Memantine augmentation in treatment-resistant obsessive-compulsive disorder: an open-label trial. J Clin Psychopharmacol.

[14] Mancuso E, Faro A, Joshi G, Geller DA (2010). Treatment of pediatric obsessive-compulsive disorder: a review. J Child Adolesc Psychopharmacol.

[15] Carlsson ML (1998). Hypothesis: is infantile autism a hypoglutamatergic disorder? Relevance of glutamate – serotonin interactions for pharmacotherapy. J Neural Transm.

[16] McDougle CJ, Kem DL, Posey DJ (2002). Case series: use of ziprasidone for maladaptive symptoms in youths with autism. J Am Acad Child Adolesc Psychiatry.

[17] Chakrabarty K, Bhattacharyya S, Christopher R, Khanna S (2005). Glutamatergic dysfunction in OCD. Neuropsychopharmacology.

[18] Coric V, Milanovic S, Wasylink S, Patel P, Malison R, Krystal JH (2003). Beneficial effects of the antiglutamatergic agent riluzole in a patient diagnosed with obsessive-compulsive disorder and major depressive disorder. Psychopharmacology (Berl).

[19] Coric V, Taskiran S, Pittenger C, Wasylink S, Mathalon DH, Valentine G (2005). Riluzole augmentation in treatment-resistant obsessive-compulsive disorder: an open-label trial. Biol Psychiatry.

[20] Pasquini M, Biondi M (2006). Memantine augmentation for refractory obsessive-compulsive disorder. Prog Neuropsychopharmacol Biol Psychiatry.

[21] Feusner JD, Kerwin L, Saxena S, Bystritsky A (2009). Differential efficacy of memantine for obsessive-compulsive disorder vs. generalized anxiety disorder: an open-label trial. Psychopharmacol Bull.

[22] Stewart SE, Jenike EA, Hezel DM, Stack DE, Dodman NH, Shuster L (2010). A single-blinded case-control study of memantine in severe obsessive-compulsive disorder. J Clin Psychopharmacol.

[23] Kolevzon A, Mathewson KA, Hollander E (2006). Selective serotonin reuptake inhibitors in autism: a review of efficacy and tolerability. J Clin Psychiatry.

[24] Bethea TC, Sikich L (2007). Early pharmacological treatment of autism: a rationale for developmental treatment. Biol Psychiatry.

[25] Williams K, Wheeler DM, Silove N, Hazell P. Selective serotonin reuptake inhibitors (SSRIs) for autism spectrum disorders (ASD). Cochrane Database Syst Rev. 2010(8):Cd004677. doi: 10.1002/14651858.CD004677.pub2. 10.1002/14651858.CD004677.pub2 20687077

[26] Naaijen J, Lythgoe DJ, Amiri H, Buitelaar JK, Glennon JC (2015). Fronto-striatal glutamatergic compounds in compulsive and impulsive syndromes: a review of magnetic resonance spectroscopy studies. Neurosci Biobehav Rev.

[27] Purkayastha P, Malapati A, Yogeeswari P, Sriram D. A review on GABA/glutamate pathway for therapeutic intervention of ASD and ADHD. Curr Med Chem. 2015 Feb 9. (Epub ahead of print). 10.2174/0929867322666150209152712 25666800

[28] Ghanizadeh A (2013). Increased glutamate and homocysteine and decreased glutamine levels in autism: a review and strategies for future studies of amino acids in autism. Dis Markers.

[29] Chez MG, Burton Q, Dowling T, Chang M, Khanna P, Kramer C (2007). Memantine as adjunctive therapy in children diagnosed with autistic spectrum disorders: an observation of initial clinical response and maintenance tolerability. J Child Neurol.

[30] Owley T, Salt J, Guter S, Grieve A, Walton L, Ayuyao N (2006). A prospective, open-label trial of memantine in the treatment of cognitive, behavioral, and memory dysfunction in pervasive developmental disorders. J Child Adolesc Psychopharmacol.

[31] Erickson CA, Posey DJ, Stigler KA, Mullett J, Katschke AR, McDougle CJ (2007). A retrospective study of memantine in children and adolescents with pervasive developmental disorders. Psychopharmacology (Berl).

[32] Findling RL, McNamara NK, Stansbrey RJ, Maxhimer R, Periclou A, Mann A (2007). A pilot evaluation of the safety, tolerability, pharmacokinetics, and effectiveness of memantine in pediatric patients with attention-deficit/hyperactivity disorder combined type. J Child Adolesc Psychopharmacol.

[33] Manolio TA, Brooks LD, Collins FS (2008). A HapMap harvest of insights into the genetics of common disease. J Clin Invest.

[34] Rotge JY, Aouizerate B, Tignol J, Bioulac B, Burbaud P, Guehl D (2010). The glutamate-based genetic immune hypothesis in obsessive-compulsive disorder. An integrative approach from genes to symptoms. Neuroscience.

[35] Moy SS, Nadler JJ (2008). Advances in behavioral genetics: mouse models of autism. Mol Psychiatry.

[36] Barnby G, Abbott A, Sykes N, Morris A, Weeks DE, Mott R (2005). Candidate-gene screening and association analysis at the autism-susceptibility locus on chromosome 16p: evidence of association at GRIN2A and ABAT. Am J Hum Genet.

[37] Arnold PD, Macmaster FP, Richter MA, Hanna GL, Sicard T, Burroughs E (2009). Glutamate receptor gene (GRIN2B) associated with reduced anterior cingulate glutamatergic concentration in pediatric obsessive-compulsive disorder. Psychiatry Res.

[38] Gottesman II, Gould TD (2003). The endophenotype concept in psychiatry: etymology and strategic intentions. Am J Psychiatry.

[39] Rommelse NN, Arias-Vasquez A, Altink ME, Buschgens CJ, Fliers E, Asherson P (2008). Neuropsychological endophenotype approach to genome-wide linkage analysis identifies susceptibility loci for ADHD on 2q21.1 and 13q12.11.. Am J Hum Genet.

[40] Mier D, Kirsch P, Meyer-Lindenberg A (2010). Neural substrates of pleiotropic action of genetic variation in COMT: a meta-analysis. Mol Psychiatry.

[41] Munafo MR, Brown SM, Hariri AR (2008). Serotonin transporter (5-HTTLPR) genotype and amygdala activation: a meta-analysis. Biol Psychiatry.

[42] Goodman WK, Lydiard RB (2007). Recognition and treatment of obsessive-compulsive disorder. J Clin Psychiatry.

[43] Rommelse NN, Franke B, Geurts HM, Hartman CA, Buitelaar JK (2010). Shared heritability of attention-deficit/hyperactivity disorder and autism spectrum disorder. Eur Child Adolesc Psychiatry.

[44] Maina G, Rosso G, Zanardini R, Bogetto F, Gennarelli M, Bocchio-Chiavetto L (2010). Serum levels of brain-derived neurotrophic factor in drug-naive obsessive-compulsive patients: a case-control study. J Affect Disord.

[45] Behl A, Swami G, Sircar SS, Bhatia MS, Banerjee BD (2010). Relationship of possible stress-related biochemical markers to oxidative/antioxidative status in obsessive-compulsive disorder. Neuropsychobiology.

[46] Palmieri L, Persico AM (2010). Mitochondrial dysfunction in autism spectrum disorders: cause or effect?. Biochim Biophys Acta.

[47] Emanuele E, Orsi P, Barale F, di Nemi SU, Bertona M, Politi P (2010). Serum levels of vascular endothelial growth factor and its receptors in patients with severe autism. Clin Biochem.

[48] Russell VA. Overview of animal models of attention deficit hyperactivity disorder (ADHD). In: Crawley JN, et al., editors. Current protocols in neuroscience. Chapter 9:Unit9.35. 2011. 10.1002/0471142301.ns0935s54 21207367

[49] van der Kooij MA, Glennon JC (2007). Animal models concerning the role of dopamine in attention-deficit hyperactivity disorder. Neurosci Biobehav Rev.

[50] Andersen SL, Greene-Colozzi EA, Sonntag KC (2010). A novel, multiple symptom model of obsessive-compulsive-like behaviors in animals. Biol Psychiatry.

[51] Marin JC, Moura PJ, Cysneiros RM, Colugnati DB, Cavalheiro EA, Scorza FA (2008). Temporal lobe epilepsy and social behavior: an animal model for autism?. Epilepsy Behav.

[52] Scahill L, Riddle MA, McSwiggin-Hardin M, Ort SI, King RA, Goodman WK (1997). Children’s Yale-Brown Obsessive Compulsive Scale: reliability and validity. J Am Acad Child Adolesc Psychiatry.

[53] Guy W (1976). Clinical global impression scale. The ECDEU Assessment Manual for Psychopharmacology-Revised Volume DHEW Publ No ADM 76.

[54] National Institute of Mental Health (1985). Special feature: rating scales and assessment instruments for use in pediatric psychopharmacology research. Psychopharmacol Bull.

[55] Shaffer D, Fisher P, Lucas CP, Dulcan MK, Schwab-Stone ME (2000). NIMH Diagnostic Interview Schedule for Children Version IV (NIMH DISC-IV): description, differences from previous versions, and reliability of some common diagnoses. J Am Acad Child Adolesc Psychiatry.

[56] Lord C, Rutter M, Le Couteur A (1994). Autism Diagnostic Interview-Revised: a revised version of a diagnostic interview for caregivers of individuals with possible pervasive developmental disorders. J Autism Dev Disord.

[57] March JS, Karayal O, Crisman A. The pediatric adverse event rating scale. Proceedings of the 54th Annual Meeting of the American Academy of Child and Adolescent Psychiatry. 2007.

[58] Aman MG, Burrow WH, Wolford PL (1995). The Aberrant Behavior Checklist-Community: factor validity and effect of subject variables for adults in group homes. Am J Ment Retard.

[59] Aman MG, Singh NN, Stewart AW, Field CJ (1985). The aberrant behavior checklist: a behavior rating scale for the assessment of treatment effects. Am J Ment Defic.

[60] Aman MG, McDougle CJ, Scahill L, Handen B, Arnold LE, Johnson C (2009). Medication and parent training in children with pervasive developmental disorders and serious behavior problems: results from a randomized clinical trial. J Am Acad Child Adolesc Psychiatry.

[61] Hartman C, Luteijn E, Moorlag H, de Bildt A, Minderaa R (2008). VISK, herziene handleiding 2007. Vragenlijst voor inventarisatie van sociaal gedrag van kinderen.

[62] Hartman CA, Luteijn E, Serra M, Minderaa R (2006). Refinement of the Children’s Social Behavior Questionnaire (CSBQ): an instrument that describes the diverse problems seen in milder forms of PDD. J Autism Dev Disord.

[63] Luteijn E, Minderaa R, Jackson S (2002). Vragenlijst voor Inventarisatie van Sociaal gedrag bij Kinderen, Handleiding [Children’s social behaviour questionnaire, manual].

[64] Bodfish JW, Symons FJ, Parker DE, Lewis MH (2000). Varieties of repetitive behavior in autism: comparisons to mental retardation. J Autism Dev Disord.

[65] Shaffer D, Gould MS, Brasic J (1983). A children’s global assessment scale (cgas). Arch Gen Psychiatry.

[66] Achenbach TM, Rescorla L (2001). ASEBA School-Age Forms & Profiles.

[67] Achenbach TM (1991). Manual for the Teacher’s Report Form and 1991 profile.

[68] Riley AW, Forrest CB, Starfield B, Rebok GW, Robertson JA, Green BF (2004). The Parent Report Form of the CHIP-Child Edition: reliability and validity. Med Care.

[69] Bralten J, Arias-Vasquez A, Makkinje R, Veltman JA, Brunner HG, Fernandez G (2011). Association of the Alzheimer’s gene SORL1 with hippocampal volume in young, healthy adults. Am J Psychiatry.

[70] Wechsler D (1991). Manual for the Wechsler intelligence scale for Children – 3rd edition (WISC-III).

[71] Crawford JR, Anderson V, Rankin PM, MacDonald J (2010). An index-based short-form of the WISC-IV with accompanying analysis of the reliability and abnormality of differences. Br J Clin Psychol.

[72] European Medicines Agency (2006). ICH Topic E 2 A – Clinical safety data management: definitions and standards for expedited reporting (CPMP/ICH/377/95).

[73] Glennon J, Purper-Ouakil D, Bakker M, Zuddas A, Hoekstra P, Dittmann RW (2014). Paediatric European Risperidone Studies (PERS): context, rationale, objectives, strategy, and challenges. Eur Child Adolesc Psychiatry.

